# Repeated iodine-125 seed implantations combined with external beam radiotherapy for the treatment of locally recurrent or metastatic stage III/IV non-small cell lung cancer: a retrospective study

**DOI:** 10.1186/s13014-016-0688-5

**Published:** 2016-09-13

**Authors:** Wei Li, Gang Dan, Jianqing Jiang, Yifeng Zheng, Xiushan Zheng, Dan Deng

**Affiliations:** Department of Thoracic Surgery, PLA Chengdu Military General Hospital, Chengdu, 610083 People’s Republic of China

**Keywords:** Non-small cell lung cancer, I^125^ seed implantation, External beam radiotherapy, Metastasis, Recurrence, Brachytherapy

## Abstract

**Background:**

Recurrent or metastatic lung cancer is difficult to manage. This retrospective study aimed to assess the efficacy of repeated iodine-125 seed implantations combined with external beam radiotherapy (EBRT) for locally recurrent or metastatic stage-III/IV non-small cell lung cancer (NSCLC).

**Methods:**

Eighteen previously treated stage-III/IV NSCLC patients with local or metastatic recurrences underwent 1-to-3 iodine-125 implantations. Six of these patients received palliative EBRT and six patients received combined chemotherapy using gemcitabine and cisplatin. Near-term treatment efficacy was evaluated 3 months after seed implantation by comparing changes in tumor size on computed tomography images; the evaluated outcomes were complete response, partial response, stable disease, and local tumor control rate. Long-term efficacy was assessed based on 1- and 2-year survival rates.

**Results:**

Patients were followed up for 6 to 50 months. The overall (i.e., complete + partial) response rate was 87.4 %. The local control rates after the first, second, and third years were 94.1, 58.8 and 41.2 %, respectively.

**Conclusions:**

The results of this study demonstrated that repeated implantation of radioactive particles combined with EBRT is a safe treatment that effectively controlled local recurrence and metastasis of stage III/IV NSCLC.

## Introduction

Lung cancer is the leading cause of cancer death in both men and women worldwide, and its prevalence is increasing [1]. Conventional treatment for lung cancer includes palliative radiotherapy and chemotherapy, which has shown limited potential in increasing long-term survival rates [2]. Using external beam radiotherapy (EBRT) alone, it is difficult to deliver a sufficient radiation dose to patients with large or advanced tumors, in order to avoid damage to adjacent normal tissues [3]. Repeated EBRT has limited efficacy for these patients and is associated with increased side effects because of the low tolerance of the surrounding normal tissue [4]. Furthermore, because a large proportion of lung cancers are diagnosed at an advanced stage, the main challenges to treatment are recurrence and metastasis, which is difficult to manage. Extensive resection of a locally recurrent lung cancer is associated with high recurrence risk and significant mortality. Mediastinal metastasis and metastasis in the brain, bone, or distant organs are common in patients with advanced lung cancer [5]. Controlling the size of these metastatic tumors may help to improve long-term survival rate.

Radioactive seed implantation, which is a form of brachytherapy, can be performed repeatedly. It has been successfully applied for treating inoperable solitary lung cancers, while avoiding excessive radiation exposure to surrounding tissues [6–8]. Clinically, brachytherapy using iodine-125 seed implantation is capable of delivering a sufficient dose of radiation to the tumor mass. However, in many recurrent and metastatic lung cancers, important organs, large vessels, or bone structures often block the pathway to the lesions, making these tumors inaccessible to needle puncture, and leading to an unsatisfactory distribution of the implanted iodine-125 seeds [9, 10]. In addition, brachytherapy would become very risky when conducting implantation in metastatic lymph nodes located near the mediastinal macrovascular area.

In this retrospective study, we investigated the efficacy of combining iodine-125 seed implantations with EBRT for salvage treatment of advanced lung cancers. Eighteen recurrent advanced non-small cell lung cancer (NSCLC) patients who previously underwent iodine-125 seed implantations received computed tomography (CT)-guided iodine-125 seed reimplantations in combination with EBRT.

## Materials and methods

### Patients

The Institutional Review Board and Ethics Committee of General Hospital of Chengdu Military Region of the PLA (#08-00125) approved this study. All patients provided written informed consent.

Between October 2006 and September 2014, 18 patients with recurrent or metastatic stage III/IV NSCLC (Table 1) underwent CT-guided iodine-125 seed reimplantations, with or without EBRT, at the General Hospital of Chengdu Military Region of PLA. All of the 18 patients enrolled in this study had a history of iodine-125 seed implantations and met the following criteria: NSCLC was confirmed by histopathological examination; a CT scan indicated a solid mass or nodule in the lung area, or nearby the mediastinum; local recurrence or restricted metastasis at <3 regions; the Karnofsky Performance Status score was ≥60.

**Table 1:** Clinical features of the present 18-patient cohort

No.	Age, y	Gender	TNM	KPS ^a^	Tumors	Size, cm^3^	Seeds ^b^; activity, mCi	MPD, Gy
1	72	M	T3N1M0	70	L, lung SCC	6 × 5 × 5	72/59/43; 0.7–0.8	130
2	57	F	T3N1M0	80	R, lower lung adenocarcinoma	6 × 6 × 5	76/45/52; 0.8	140
3	59	M	T3N1M1	70	L, lower lung adenocarcinoma	6 × 5 × 4	77/20; 0.7	140
4	59	M	T3N2M0	70	L, lung SCC	5 × 5 × 4	66/62; 0.8	150
5	73	M	T2N2M0	80	R, lower lung adenocarcinoma with mediastinal LNM	6 × 7 × 6	63/24; 0.8	140
6	70	M	T3N1M1	70	L, lung SCC with brain metastases	5 × 5 × 4	45; 0.7	130
7	54	M	T3N1M0	60	L, lung poorly differentiated SCC	8 × 7 × 5	100/40; 0.6	150
8	81	M	T3N1M0	70	R, lower lung adenocarcinoma with vertebral metastases	6 × 5 × 5	65/45; 0.8	140
9	66	F	T4N1M0	80	R, lower lung SCC	6 × 5 × 4	70/62; 0.7	130
10	67	M	T2N1M1	70	R, upper lung SCC with LNM	4 × 4 × 3	48/20/14; 0.8	130
11	73	M	T2N1M0	60	L, lung SCC in situ recurrence after 1 y	5 × 5 × 4	62/40; 0.8	140
12	78	F	T2N0M0	70	R, upper lung squamous	3 × 1 × 2	23/40; 0.7	140
13	65	F	T4N2M0	70	SCC with mediastinal lymph node metastases	7 × 6 × 6	89; 0.7	130
14	75	M	T3N1M0	60	Adenocarcinoma	5 × 6 × 5	19/40; 0.7	130
15	66	M	T3N2M1	80	R, upper lung adenocarcinoma with vertebral metastases	7 × 5 × 4	78; 0.6	160
16	60	F	T2N1M1	70	R, upper lung adenocarcinoma with adrenal metastasis	6 × 5 × 5	70; 0.8	140
17	57	M	T4N1M1	75	L, lung SCC associated with vertebral metastases	5 × 5 × 3	41/36; 0.8	130
18	64	M	T3N2M0	80	R, lung moderately differentiated SCC with mediastinal LNM	4 × 5 × 5	52; 0.8	120

*L* left, *LR* local recurrence, *LNM* lymph node metastasis, *MPD* matched peripheral dose, *R* right, *SCC* squamous cell carcinoma, *TNM* tumor, node, metastasis stage

^a^ KPS score; ^b^ number of iodine-125 seeds per implantation

### Treatment

Among the 18 patients evaluated (Tables 1 and 2), two patients with vertebral metastasis (Patients 8 and 17) and great pain and one patient with metastasis in the lymph nodes of the mediastinum (Patient 13) received palliative EBRT at 20–30 Gy. In addition, three patients with mediastinum metastasis (Patients 5, 10, and 18) received palliative EBRT at the lymph nodes of the mediastinum. Two patients with brain metastasis (Patients 3 and 6) underwent gamma knife radiosurgery.

**Table 2:** Recurrence and survival of the present 18-patient cohort

No.	Age, y	Gender	Recurrence/metastasis after first seed implant, further treatment	TTR mo	Survival, mo
1	72	M	LR; 3 implants	11/12	43
2	57	F	LR; 3 implants	10/13	31
3	59	M	Brain metastases; whole brain irradiation + 2 implants	24	Living
4	59	M	Adrenal metastasis (R); 2 implants	13	Living
5	73	M	LR; 2 implants, yet failed due to hemoptysis	15	15
6	70	M	—	12	Living
7	54	M	LR; 2 implants + mediastinal external irradiation	11	13
8	81	M	—	14	24
9	66	F	LR; 2 implants	17	Living
10	67	M	Neck metastasis; 3 implants	11/13	17
11	73	M	LR; 2 implants	14	Living
12	78	F	L; lung metastases at 6 years after first treatment; 2 implants	78	Living
13	65	F	—	0	Living
14	75	M	Neck metastasis; 2 implants	13	16
15	66	M	—	16	21
16	60	F	Neck metastasis; 1 implant + adrenal EBRT	0	11
17	57	M	—	0	12
18	64	M	—	0	Living

*LR* local recurrence, *TTR* time to recurrence

Seven patients (Patients 2, 5, 7, 11, 14, 16, and 18) received combined chemotherapy using gemcitabine and cisplatin. The regimen had a 21-day schedule during which gemcitabine (1000 mg/m^2^) was administered on the first and eighth days, and cisplatin (20 mg/m^2^) on the first, second, and third days. The chemotherapy schedule was repeated for 4 to 6 cycles, if tolerated.

All patients received iodine-125 implantations. One-to-2 weeks before iodine-125 seed implantation, routine blood examination, bleeding time, and coagulation tests were performed to exclude contraindications for needle puncture.

Routine enhanced-CT scans were also performed for assessing tumor volume. Briefly, gross tumor volume was outlined and used as the planning target volume. The minimum matched peripheral dose was set at 110–140 Gy. All the implantations were performed in a standard CT room under local anesthesia, and guided by CT using a Fudan TPS 2.00 brachytherapy planning system [11] (Table 1). Iodine-125 seeds with a nominal activity of 0.5–0.7 millicurie (mCi) per seed and a diameter <1 mm were implanted using a turntable implantation gun with 18-G implantation needles (XinKe Pharmaceutical, Shanghai, China), while avoiding puncturing of the nearby vessels and other organs. Patients were kept in the radiation oncology/interventional ward for 1-to-2 days after implantation.

The distribution of the radioactive seeds was evaluated immediately after implantation by CT scans. Re-implantation was conducted for sites showing an uneven distribution of seeds. The minimum peripheral dose was 110–140 Gy (mean, 120 Gy).

### Follow-up and efficacy evaluation

Each patient underwent a follow-up examination at 1 and 3 months after the seed implantation, and then every 3 months for up to 60 months. Physical examinations, blood tests, and thoracic CT scans were performed. Patients’ pain score, the rate of radiation pneumonia, time to recurrence, survival and local control rates, and median survival times were recorded. Survival and locoregional metastasis rates were calculated using the Kaplan-Meier method. For calculation of the survival rate, deaths from any cause were scored as events. Local control was defined as lack of tumor progression in areas adjacent to or at the site of iodine-125 seed implantation and adjacent regions.

To evaluate near-term efficacy after brachytherapy, all patients underwent CT scans 3 months after seed implantation. The total volume of each tumor was normalized to that before implantation. Complete response was defined as the complete disappearance of a lesion for >4 weeks. A partial response was considered when the size of the lesion decreased by >50 %, and then remained unchanged for 4 weeks. Stable disease was defined when the size of the tumor decreased by <50 % or increased by <25 %. Response rate was defined as the sum of the complete response and partial response. Local tumor control was defined as the absence of tumor progression detected by CT (i.e., stable disease + partial response + complete response). Long-term efficacy was assessed according to 1-year and 2-year survival rates.

## Results

### Efficacy

Eighteen patients with recurrent and metastatic advanced NSCLC received a total of 35 implantations of iodine-125 seeds (Table 1). Based on the imaging results obtained at approximately 2 months after the first implantation, near-term complete response was achieved in seven cases, partial response in another six cases, and stable disease in five cases. The overall response rate was 72.22 % (i.e., 13/18).

During follow-up after the first round of iodine-125 seed implantation, 12 of the 18 patients experienced tumor recurrence and underwent a second round of iodine-125 seed implantations (Table 2). Four of these 13 patients (Nos. 1, 2, 3, and 10) experienced tumor recurrence again, and underwent a third round of seed implantations. Metastatic tumors were found in the adrenal glands of patient No. 16, and in the vertebra of patient Nos. 8 and 10.

For patient Nos. 6, 8, 13, 15, 17 and 18, the residual tissues within each tumor mass showed no enhancement on the CT or positron emission tomography (PET)-CT images obtained 3 months after the first round of iodine-125 seed implantation (Table 2). In eight patients, PET-CT scans detected reduced metabolic activity in the lesions or disappearance of the lesions and residual tissues within the tumor mass.

**Fig. 1:**
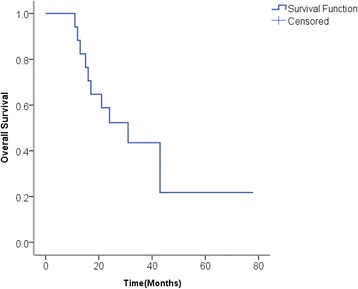
Kaplan-Meier curve of 18 patients with recurrent or metastatic stage III/IV non-small cell lung cancer

As calculated from the Kaplan-Meier Curve (Fig. 1), the median survival time for this 18-patient cohort was 31 months, and the progression-free survival time was 6–8 months. The overall 1-year and 2-year survival rates were 62.5 and 32.7 %, respectively.

Ten patients reported cancer-related pain before treatment. After treatment, complete relief was achieved in all patients with mild pain (*n* = 4), and in two of four patients with moderate pain. Partial relief was achieved in the other two of the four patients with moderate pain, and in one of two patients with severe pain. One patient with severe pain showed no relief. The three patients with partial or no pain relief received steroid-based painkillers.

Two patients (Nos. 7 and 14) experienced rapid tumor recurrence and metastasis in multiple sites after the second implantation, and died of multiple organ failure thereafter.

### Adverse reactions and complications

Complications include pneumothorax and hemoptysis, both are common to puncture. One patient had severe hemoptysis.

The most common complications reported were pain, pneumothorax, and hemoptysis, which are common to puncture. One patient developed grade 2 or higher radiation pneumonitis after the first round of iodine-125 seed implantation. Three patients experienced pneumonitis after the second round of iodine-125 seed implantation. One patient (No. 5) with squamous cell lung carcinoma in the lower right lung experienced tumor recurrence 18 months after the first round of seed implantation. During the puncture process of the second round of seed implantation, the patient developed severe hemoptysis and died of respiratory failure 3 days later. All the other patients underwent no radiation pneumonitis or other serious complications after the second round of iodine-125 seed implantation.

### Typical cases

**Fig. 2:**
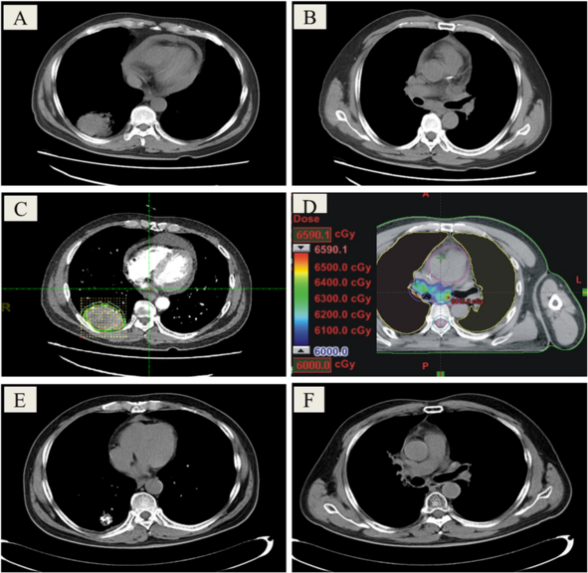
CT images of moderately differentiated squamous cell carcinoma (4 × 5 × 5 cm^3^) in the lower right lung (**a**) with metastasis in the lymph nodes of the mediastinum (**b**) in a 64-year old man (No. 18). The patient received iodine-125 seed implantation for lung tumor (**c**) and external beam radiotherapy for lymph node metastasis (**d**). Complete disappearance of the lung tumor (**e**) and lymph node metastasis (**f**) was observed 1 month after treatment

A 64-year old man (No. 18) with moderately differentiated squamous cell carcinoma (4 × 5 × 5 cm^3^) in the lower right lung and metastasis in the lymph nodes of the mediastinum showed complete response to the combined therapy (Fig. 2). Iodine-125 seeds were implanted to his lung tumor region for brachytherapy; EBRT was used to treat lymph node metastasis. The patient showed complete disappearance of the lung tumor, 2 months after implantation. He developed no radiation pneumonia. He was tumor-free for 2 years and is still living.

**Fig. 3:**
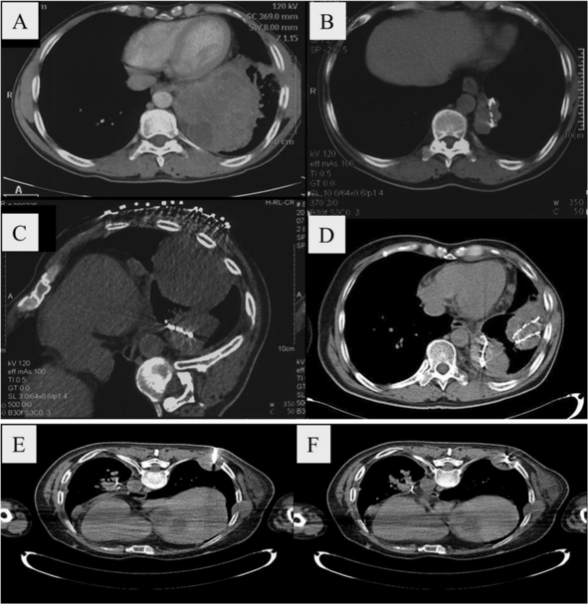
CT images of a squamous cell lung carcinoma in the lower left lung in a 56-year-old man (No. 3) with brain metastasis: (**a**) before treatment; (**b**) 12 months after the first iodine-125 seed implantation of iodine-125 seeds; (**c**) 30 months after the first implantation showing a recurrent tumor mass (8 × 4 cm^2^) in the lower left thoracic cavity; (**d**) 33 months after the first, i.e. 3 months after the second implantation, showing that the tumor size had decreased by 50 %; (**e**) during the third implantation, i.e., 42 months after the first implantation, showing metastasis on the right side of the chest wall; (**f**) Immediately after the third implantation

A 56-year-old man (No. 3) with squamous cell lung carcinoma in the lower left lung and brain metastasis exhibited a significant reduction in tumor size after the first implantation of iodine-125 seeds (Fig. 3). Brain EBRT effectively provided control of the metastasis, while the patient also received 6 cycles of chemotherapy. Tumor size gradually reduced during follow-up and the CT image was tumor-free at the 12-month follow-up. Thirty months after the first round of brachytherapy, a recurrent tumor mass (8 × 4 cm^2^) was detected in the lower left thoracic cavity and the patient underwent a second seed implantation procedure. Three months later, the tumor size was reduced by 50 %. Forty-two months after the first brachytherapy, metastasis was found on the right side of the chest wall, and the patient received seed implantation for the third time and lived for 4 months afterwards.

**fig. 4:**
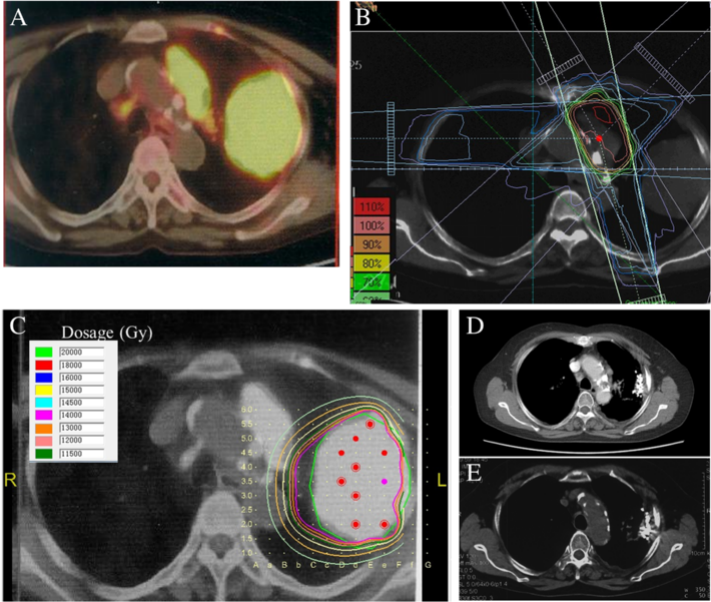
PET-CT image of a massive squamous cell carcinoma (7 × 5 cm^2^) in the upper left lung with metastasis in the superior border of the pericardium and mediastinum (3 × 4 cm^2^) in a 65-year-old woman (No. 13) (**a**). The metastasis region was treated with EBRT at a dose of 30 Gy (**b**) and the massive lung cancer region was implanted with iodine-125 seeds (red and purple dots) for brachytherapy (**c**) guided by CT. Thoracic CT image was tumor-free at 1-year follow-up (**d**), and showed no recurrence of cancer at 3-year follow-up (**e**)

A 65-year-old woman (No. 13) had squamous cell lung carcinoma in the upper left lung (7 × 5 cm^2^) and metastasis in the superior border of the pericardium and mediastinum (3 × 4 cm^2^) (Fig. 4). Due to the large volume of the tumor in the upper left lung, it was hard to keep the volume of normal lung tissue that receiving a radiation dose of 20 Gy (V20) under 30 % when conducting EBRT. Moreover, it was risky to conduct a puncture of the tumor in the mediastinum due to its proximity to large blood vessels. Therefore, seed particles were implanted in the lung tumor while EBRT was delivered to the mediastinum tumor at a dose of 30 Gy. One year after implantation, complete response was achieved. Three years after implantation, no recurrence was observed.

**Fig. 5:**
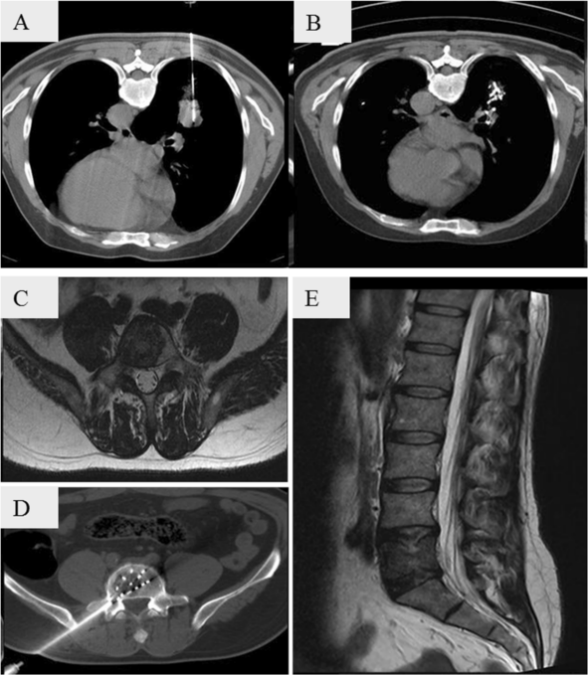
CT images showing brachytherapy of non-small cell lung adenocarcinoma (3 × 2 cm^2^) with vertebral metastasis in a 57-year-old man (No. 17). Both the adenocarcinoma region in the lower right lung (**a**) and the vertebral metastasis region (**c, d**) were implanted with iodine-125 seeds. One-year follow-up images showed good tumor control in both regions (**b, e**)

A 57-year-old man (No. 17) had an adenocarcinoma cell lung carcinoma in the lower right lung (3 × 2 cm^2^) and metastasis in the vertebra (Fig. 5). Iodine-125 seeds were implanted in both regions and the patient survived for 15 months without further metastasis in the vertebra.

## Discussion

Stage III/IV NSCLC patients with recurrence and metastasis are often inoperable. Palliative radiotherapy and chemotherapy are recommended for these patients, but the efficacy of EBRT and chemotherapy is very limited and the survival time is often short (e.g., 6–9 months). In the present study, EBRT was combined with iodine-125 seed implantations for treating locally recurrent NSCLC with concurrent metastasis (patient Nos. 6, 13, 15, 17 and 18). The iodine-125 seed implantations were delivered, at enough radiation doses, to tumors that were difficult to reach by EBRT. We found that repeated iodine-125 seed implantations were beneficial for patients with multiple recurrences, leading to longer median survival time and progression-free survival time, and higher overall 1-year and 2-year survival rates.

For tumors >5 cm, it is difficult to deliver a large enough dose with EBRT in order to avoid damaging adjacent normal tissues such as the lung, spinal cord, and heart [12]. It has been reported that the effective EBRT dose for tumors >5 cm is greater than 100 Gy [3]. However, this high dose of EBRT can lead to an increase in V20 (i.e., more than 30 % of the surrounding normal lung tissue receiving a radiation dose of 20 Gy), thereby increasing the risk of complications. Thus, low doses of EBRT (30–40 Gy) are typically delivered for palliative treatments. In contrast, iodine-125 seed implantation has the potential to deliver a higher radiation dose to a tumor mass (100–140 Gy), and this dose drops off sharply within a short distance. Consequently, adjacent normal tissues are exposed to a minimal dose of radiation, and the short-term risk for serious radiation-induced pneumonitis is reduced [3]. In addition, compared with EBRT, the delivered dose to a tumor by iodine-125 seed implantation can be as much as 2-fold higher [13], with superior treatment efficacy [14]. The response rate reported for EBRT for advanced lung cancer is low, usually below 60 % [14]. In the present study, the response rate for iodine-125 seed implantation was 88 %, consistent with the rates reported in previous studies [6, 15].

There are limitations associated with iodine-125 seed implantation. First, it is difficult to perform needle puncture for a metastasis close to the mediastinum, and this may result in an unsatisfactory distribution of iodine-125 seeds. Similarly, needle punctures of metastases in the brain or in the lymph nodes of the mediastinum are problematic because of to their proximity to large blood vessels, the heart, or bone structures. The efficacy of iodine-125 seed implantations can also be limited by many factors, such as shielding by bone structures, differences in brachytherapy planning systems, and individual differences. Generally, iodine-125 seed implantation is an effective treatment for large tumors. However, its efficacy for poorly differentiated tumors such as small cell lung cancer is poor, due to the low initial dose of iodine-125 that is applied. Thus, protactinium-103, which has a higher initial dose, may be more effective for poorly differentiated tumors such as small cell lung cancer.

Iodine-125 seed implantation in combination with EBRT appears to control recurrent lung cancer and metastasis effectively. For example, the progression-free survival time for the present cohort was 6–8 months, the 1 and 2-year survival rates were 62.5 and 32.7 %, and middle survival time of 18 patients was 31 months, respectively. Moreover, these results are an improvement over that have been previously reported [16, 17]. One reason for this may be because the combined iodine-125 seed implantation and EBRT provide a better control of the late-stage recurrent lung cancers and metastases in vital organs by increasing the cumulative radiation dose. Another reason may be because that all of in the present study the patients included only experienced restricted metastasis, and patients with diffusive or multiple metastasis were excluded.

## Conclusions

The results of this study demonstrated that iodine-125 seed implantation in combination with EBRT has the potential to effectively control recurrent and metastatic lung cancer, with less invasiveness, fewer side effects, and higher survival rates. For tumors that are in proximity of the mediastinum or large blood vessels, and thus are inaccessible to puncture, additional EBRT was also beneficial. Thus, iodine-125 seed implantation in combination with EBRT should be considered an alternative therapy for advanced-stage NSCLC patients. Further studies are needed to confirm this treatment.

## Abbreviations

CT: Computed tomography
EBRT: External beam radiotherapy, EBRT
NSCLC: Non-small cell lung cancer
PET: Positron emission tomography
